# Acute and chronic traumatic diaphragmatic hernia: 10 years’ experience

**DOI:** 10.1371/journal.pone.0226364

**Published:** 2019-12-12

**Authors:** Pengcheng Gu, Yang Lu, Xigong Li, Xiangjin Lin

**Affiliations:** 1 Trauma centre, First Affiliated Hospital, Zhejiang University School of Medicine, Hangzhou, China; 2 Department of Orthopedics, First Affiliated Hospital, Zhejiang University School of Medicine, Hangzhou, China; Yale University, UNITED STATES

## Abstract

Controversy persists regarding many aspects of traumatic diaphragmatic hernia (TDH). We aimed to understand why some traumatic diaphragmatic injuries present with chronic hernia and to evaluate diagnosis and treatment options. Fifty acute and 19 chronic TDH patients were diagnosed and treated at our institution over a 10-year period. Clinical data from these two groups were analyzed statistically and compared. Chronic TDH patients had a significantly lower Injury Severity Score than acute TDH patients (10.26 ± 2.68 vs. 26.92 ± 4.79, P < 0.001). The most common surgical approach for acute and chronic TDH was thoracotomy and laparotomy, respectively. The length of the diaphragmatic rupture was significantly shorter in chronic TDH patients than acute TDH patients (6.00 ± 1.94 cm vs. 10.71 ± 3.30 cm, P < 0.001). The mean length of hospital stay was significantly longer for acute TDH patients than chronic TDH patients (41.18 ± 31.02 days vs. 16.65 ± 9.61 days, P = 0.002). In conclusion, milder trauma and a smaller diaphragmatic rupture were associated with delayed diagnosis. A thoraco-abdominal computed tomography scan is needed for patients with periphrenic injuries to avoid delayed diagnosis of TDH. Improved awareness and understanding of diaphragmatic injuries will increase the rate of early diagnosis and improve prognosis.

## Introduction

Traumatic diaphragmatic hernia (TDH) is an uncommon disease, encountered in 0.8% to 6% of blunt trauma and more than 17% of thoraco-abdominal-penetrating trauma.[1–6] In the acute phase, TDH is often coupled with other thoraco-abdominal, cerebral, or musculoskeletal injuries. These more severe comorbidities, rather than the diaphragmatic injury itself, are responsible for the poor prognosis.[2, 7, 8] In the chronic phase, the hernia may cause gut obstruction or have a pathological impact on the respiratory and circulatory systems, causing high morbidity and mortality.[9]

Previous studies indicate that 2.7% to 50% of diaphragmatic ruptures may not be diagnosed initially.[10, 11] Improved awareness and the development of imaging techniques, in particular computed tomography (CT), have increased the rate of early, correct diagnosis.[12–14] Nevertheless, an unidentified rupture can enlarge when intra-abdominal pressure rises. Because of the natural pressure differential between the abdomen and thorax, and the continuous movement of the diaphragm, the rupture may not heal spontaneously.[15] Carter *et al*. noted that patients can experience an asymptomatic period, referred to as the “latent phase,” for decades before finally manifesting symptoms, sometimes with severe complications.[16]

The natural history, causes, ways to prevent delays in diagnosis, and operative approaches of TDH remain controversial. The absence of comparisons between acute and chronic TDH limits understanding of this clinical entity. Here we report our experience diagnosing and treating TDH and compare acute and chronic cases, hoping to better understand the pattern of these two clinical complaints.

## Patients and methods

This study was conducted in November 2018. All 69 TDH cases were diagnosed and treated between May 2007 and December 2017 at a single institution. This study was approved by the ethics committee of the first affiliated hospital of zhejiang university. The approval number was 2018–823. According to our ethics committee, form of consent was not needed for retrospective studies. Nineteen of the cases were chronic TDH. All patients had surgical confirmation except for two chronic TDH patients who refused surgery and were diagnosed by spiral CT. We collected clinical data, including demographic information, mechanisms and severity of the injury, clinical presentation, imaging methods, characteristics of the hernia (side, organs, length of rupture, etc.), operation approaches, length of hospital stay (LOS), and outcomes.

The Abbreviated Injury Scale (AIS) is used to assess the severity of injuries in different parts of the human body, and the Injury Severity Score (ISS) is calculated based on the AIS.[17] The ISS of chronic patients was scored according to the initial injuries. The size of the diaphragmatic ruptures and herniated organs were recorded by the operating surgeon. Diagnostic imaging studies included chest radiograph (CR) and spiral CT.

The definition of chronic TDH is controversial. Some authors use a specific time, such as 7 days or 1 month, as a cutoff for acute TDH, regardless of the time of the surgery or hospitalization.[9] We defined chronic TDH as diaphragmatic hernia with an explicit trauma history that was not diagnosed during the initial hospitalization. All patients diagnosed with chronic TDH had been to the hospital at the time of the trauma and had been discharged without a diagnosis of TDH after examination.

We had no access to information that could identify any participants during or after data collection. We performed intergroup analyses using the chi-square test or Student’s t test, and P < 0.05 was considered statistically significant. We performed all analyses and calculations using SPSS (version 25; IBM, Chicago, IL, USA).

## Results

### Clinical presentation and diagnostic studies

Of the 50 patients with acute TDH, 41 were male and nine were female (4.6:1). The mean patient age was 51 ± 14 years (13–77 years). A traffic accident (34 cases) was the most prevalent trauma mechanism, followed by an accidental fall (eight cases), penetrating injury (five cases), and bruising injury caused by a heavy object (three cases). Thirty-six patients had a left-sided hernia and 14 had a right-sided hernia (2.6:1). The mean ISS was 27 ± 5 (13–36). Diagnoses were made by CR, CT, and surgical exploration in 16, 28, and 6 patients, respectively. CT had a sensitivity of 93% and specificity of 100%. No patients with concern for diaphragm injury were confirmed negative by surgical exploration.

Of the 19 patients with chronic TDH, eight were male and 11 were female (0.7:1). The mean patient age was 52 ± 17 years (32–85 years). At the initial injury, 13 patients were asymptomatic, and six experienced slight to moderate abdominal or chest pain. In the chronic phase, 14 patients complained of abdominal discomfort, including abdominal pain (nine cases), nausea and/or vomiting (four cases), and hematemesis (one case). Two patients complained of chest tightness and chest pain. Three patients were asymptomatic. The median duration of symptoms was 7 days (1–900 days). The most prevalent trauma mechanism was a traffic accident (13 cases), followed by penetrating injury (four cases) and an accidental fall (two cases). The interval between the trauma and diagnosis was 8 ± 7 years (1–30 years). Fourteen patients had a left-sided hernia and five had a right-sided hernia (2.8:1). The mean ISS of the previous injury was 10 ± 3 (5–17). The diagnosis was confirmed by CT in all patients. The sensitivity and specificity were both 100%

### Surgical modalities and findings

All of the acute TDH patients underwent surgery to ameliorate herniated organs and repair the diaphragm by non-absorbable suture. The operative approaches were posterior thoracotomy in 26 patients, combined laparotomy and midline thoracotomy in eight patients, midline laparotomy in six patients, left subcostal laparotomy in six patients, combined posterior thoracotomy and left subcostal laparotomy in two patients, and midaxillary line thoracotomy in two patients. The mean length of the diaphragm rupture was 11 ± 3 cm (4–20 cm). The most common herniated organ was the stomach (22 cases), followed by the omentum (20 cases), liver (14 cases), colon (nine cases), spleen (eight cases), small intestine (six cases), and cholecyst (three cases).

Two chronic TDH patients rejected surgical treatment. One was asymptomatic and the other felt slightly oversatiated after meals. Seventeen patients underwent surgery to repair the diaphragm by non-absorbable suture. The operative approaches were midline laparotomy in 10 patients and posterior thoracotomy in seven patients. An additional left subcostal laparotomy was added to the posterior thoracotomy for one patient because the incarcerated and necrotic intestines needed to be excised. Two patients who underwent midline laparotomy were given additional surgery besides diaphragm repair. One patient had an additional incision decompression of the intestine, and another patient had a colostomy after resection of the left colic flexure. The mean length of the diaphragm rupture was 6 ± 2 cm (4–11 cm). The most common herniated organ was the colon (nine cases), followed by the omentum (seven cases), stomach (five cases), small intestines (three cases), liver (three cases), spleen (three cases), and cholecyst (one case).

#### Outcomes

The mean LOS was 41 ± 31 days (5–198 days) for acute TDH patients. Postoperative complications included pneumonia or atelectasis (n = 6), urinary tract infection (n = 4), septicopyemia (n = 3), infection of surgical incision (n = 2), renal failure (n = 2), ileus (n = 1), and pulmonary thromboembolism (n = 1) The death rates for left-sided and right-sided TDH patients were 8.3% (n = 3) and 14.3% (n = 2), respectively. Reasons for death were septicopyemia (n = 2), multiple organ failure (n = 1), respiratory failure due to severe pneumonia (n = 1), and hematencephalon or cerebral infarction (n = 1). The mean follow-up time was 5 ± 4 years. At the end of follow-up, only one patient had a recurrence and underwent another posterior thoracotomy to repair the re-ruptured diaphragm. Mesh was used to reduce tension.

The mean LOS was 17 ± 10 days (5–35 days) for the chronic TDH patients. The three patients who had additional surgeries other than diaphragm repair had the longest hospital stays (26, 30, and 35 days). The mean follow-up time was 6 ± 4 years, and no patient had a recurrence at the final follow-up date.

### Comparisons of acute and chronic TDH

There was a significant difference in the male/female ratio between acute and chronic TDH patients. Males accounted for 82.0% of acute TDH patients but only 42.1% of chronic patients. Acute TDH patients were mostly admitted by the emergency department, whereas chronic TDH patients were mostly admitted by the outpatient service (P < 0.001; Table 1).

**Table 1:** Comparisons between acute and chronic TDH (enumeration data).

		Acute	Chronic	P value
Age	≥60	12	5	0.842
	<60	38	14	
Gender	Female	9	11	0.001
	Male	41	8	
Admission	outpatient	5	15	<0.001
	emergency	45	4	
Side	Left	36	14	0.889
	Right	14	5	
Mechanism of trauma	Penetrating	5	4	0.223
	Blunt	45	15	
Intestines henia	Present	15	12	0.012
	Absent	35	7	
Liver hernia (right-sided)	Present	14	3	0.066
	Absent	0	2	
Surgical approaches	Thoracotomy	28	7	0.162
	Laparotomy	12	10	
	Thoraco-abdominal	10	1	

traumatic diaphragmatic hernia (TDH)

Chronic TDH patients had a significantly lower ISS than acute patients (10.26 ± 2.68 vs. 26.92 ± 4.79, P < 0.001). The length of the diaphragm rupture was significantly shorter in chronic TDH patients than acute TDH patients (6.00 ± 1.94 cm vs. 10.71 ± 3.30 cm, P < 0.001). The mean LOS was significantly longer for acute TDH patients than for chronic patients (41.18 ± 31.02 days vs. 16.65 ± 9.61 days, P = 0.002; Table 2).

**Table 2:** Comparisons of between acute and chronic TDH (measurement data).

	Acute	Chronic	P value
ISS	26.92 ± 4.79	10.26 ± 2.68	<0.001
Length of the rupture (cm)	10.71 ± 3.30	6.00 ± 1.94	<0.001
LOS (days)	41.18 ± 31.02	16.65 ± 9.61	0.002

traumatic diaphragmatic hernia (TDH)

Injury Severity Score (ISS)

length of the hospital stay (LOS)

The most common surgical approaches for acute and chronic TDH were thoracotomy and laparotomy, respectively. Intestinal hernia was more common for chronic TDH patients (P = 0.012).

## Discussion

TDH remains challenging for clinicians. The main cause of TDH largely depends on geographic and socioeconomic factors.[7, 8] At our institution, blunt trauma following high-velocity vehicular crashes is the most common cause of TDH. The different social activities that women and men participate in may contribute to the difference in sex ratios between acute and chronic TDH in our study, as men are at higher risk for more severe injuries. Left-sided TDH is more common than right-sided TDH, in particular in blunt-trauma victims, although an equal incidence is observed in patients who die before receiving definitive medical care.[1, 13, 18, 19] Bilateral injuries and tears that extend into the central tendon of the diaphragm are reported in only 2% to 6% of patients with diaphragmatic injuries.[18]

In our experience, respiratory symptoms are more prevalent for patients with acute TDH. However, it is difficult to study clinical symptoms in the acute phase, as patients may be unconscious or the symptoms may be subtle. For chronic TDH patients, gastrointestinal complaints caused by volvulus or incarceration of the intra-abdominal viscera are more common than respiratory symptoms (7:1). We also found that there was a higher rate of intestinal hernia in the chronic phase than in the acute phase. This may be attributable to the anatomical structures of the intra-abdominal organs and peristalsis of the intestines. Therefore, when patients have abdominal complaints, clinicians should refer to both recent and past trauma histories. Chronic TDH should be diagnosed promptly, because obstruction and strangulation of the intra-abdominal organs increase morbidity and mortality.

### How chronic hernia occurs

Our study shows that diaphragmatic ruptures of chronic TDH are smaller than those of acute TDH. Grimes *et al*. proposed that the rupture is enlarged during the latent phase,[20] and thus the initial ruptures should have been even smaller than what we observed during surgery. We think the small size of the rupture is the most important reason why these diaphragmatic injuries were not diagnosed initially. If the rupture is small and there is no hernia, patients will be asymptomatic and imaging studies will probably fail to observe the diaphragm injury.

It was previously thought that, in emergency situations, the symptoms of diaphragmatic hernia are obscured by other more dominant or life-threatening comorbidities. However, our results contradict this assumption. Patients with milder trauma are less likely to have a large rupture with hernia, leading to no symptoms or radiological abnormalities.

Another suggestion is that, for right-sided diaphragmatic ruptures, the liver may not herniate into the thorax in the acute phase but may continue to serve a protective role that limits protrusion of intra-abdominal organs. In our series, all cases of acute, right-sided TDH presented with liver hernia, whereas only half of the chronic TDH patients had liver hernia. This is consistent with the above theory.[4, 21, 22]

### How to prevent delayed diagnosis

**Fig 1:**
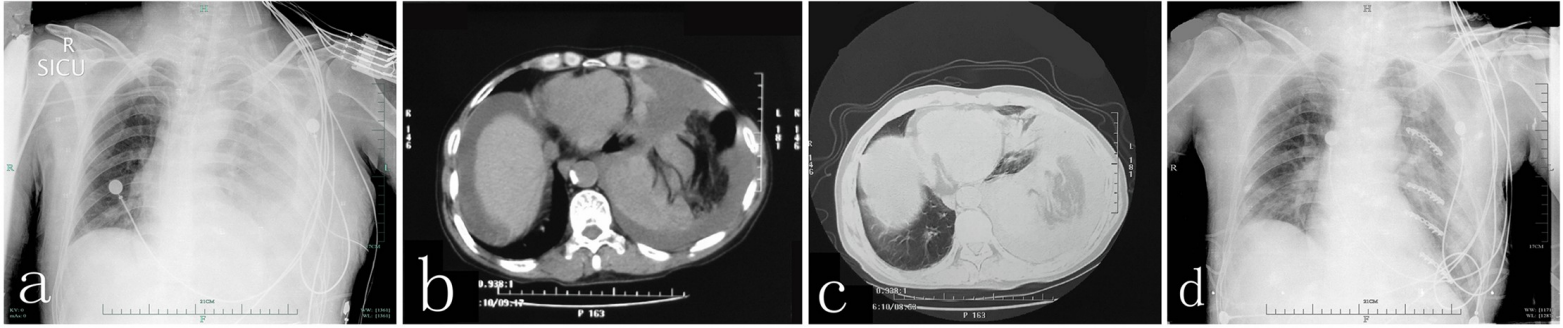
Pre-operative CR and CT (a, b and c) showed massive pleural effusion and the edge of the diaphragm was not clear; post-operative CR (d).

**Fig 2:**
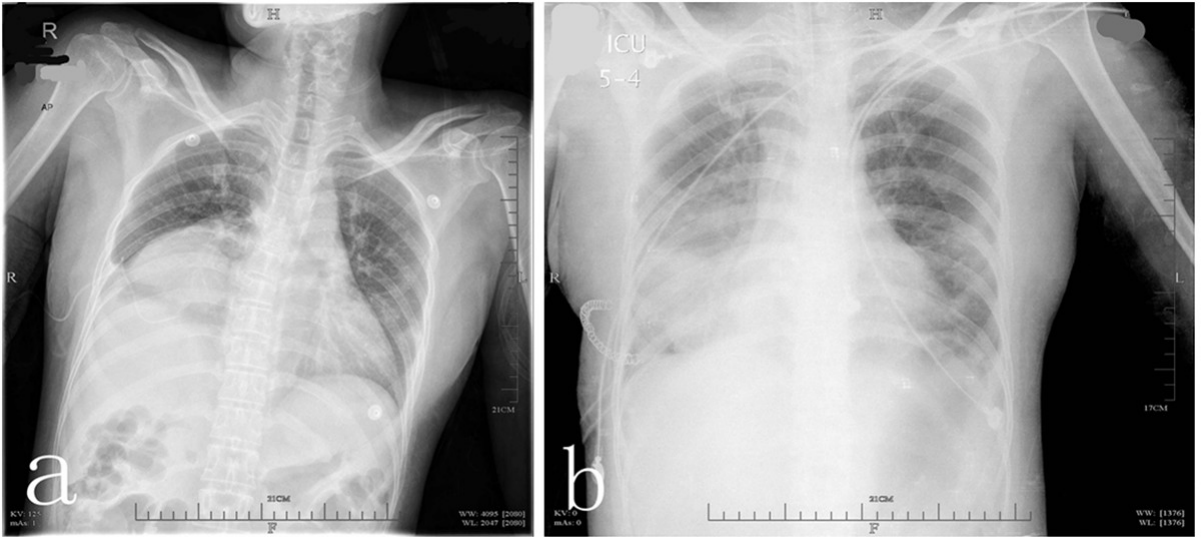
Pre-operative CR (a) showed right-sided TDH; post-operative CR (b).

Because of a variety of diagnostic pitfalls, diaphragmatic rupture is often overlooked in patients with thoracoabdominal injury.[23] In our series of patients, one 54-year-old male patient felt chest pain after a traffic accident. CR and CT showed massive pleural effusion and multiple rib fractures (Fig 1). A great quantity of bloody fluid was drained out through the chest tube, and rib fracture was the suspected cause. However, no intercostal artery damage was discovered during the surgery to repair the ribs, and substantial amounts of bloody fluid continued to drain out. During the exploratory thoracotomy, we found that the ruptured spleen herniated into the thorax. Unfortunately, this patient died of septicopyemia and multiple organ failure after 70 days in intensive care. Multiple rib fractures and pneumohemothoraxes are the most common comorbidities seen in blunt-trauma TDH patients (90%), and spleen injuries are present in 27% to 60% of cases.[13] Karmy-Jones *et al*. showed that diaphragmatic injuries cannot be excluded until extubation is achieved.[24] We also treated one 49-year-old female patient who experienced severe chest tightness right after succeeding extubation following tibiofibular internal fixation; chest X-ray suggested TDH (Fig 2). Nine of our 50 acute TDH cases were diagnosed after 24 h, with a median time of 76 h (36–288 h) after admission. The other patients were diagnosed within 24 h. TDH should not be overlooked for blunt-trauma patients with pathological changes around the diaphragm.

Although some diaphragmatic ruptures may be very difficult to diagnose initially, close cooperation between clinicians and radiologists may prevent missed diagnoses. In our study, CR was diagnostic in 32% of acute cases, which allowed for timely surgery. CT was the most common diagnostic strategy in both acute and chronic TDH patients. Previous studies indicate that admission supine CR is the most commonly performed diagnostic study after trauma and establishes a correct diagnosis in 27% to 62% blunt-trauma TDH patients. In 18% of cases, CR is suggestive but not diagnostic because of the small wound size of penetrating trauma, superimposed pathology around the diaphragm, and the protective function of the liver.[12] Many of our patients with chronic TDH could not provide an initial medical record. Therefore, we could not provide the data from the initial diagnostic studies. However, of those who were treated at our institution for the initial injury or those who knew what diagnostic studies they had received, none had received an initial CT scan. In our clinical practice, a CT scan is always needed for patients with penetrating trauma. When CR raises concern or there is suspicion of diaphragmatic, thoracic, or abdominal organ injuries, a spiral CT should always be obtained.[14] The spiral CT has improved the ability to observe breakage on the diaphragmatic muscle, even when there is no hernia. The discontinuity of the diaphragm sign, the collar sign, and the dependent viscera sign are the three most useful CT signs.[13, 25]

Despite the differences in clinical presentation between acute and chronic TDH, a careful physical examination, diagnostic imaging study, and even endoscopic exploration [3, 4] are important to discover diaphragmatic injuries initially.

### Treatment

Once the a diagnosis of TDH is made, surgery to reduce the herniated organs and repair the diaphragm is mandatory.[26] If the patient’s conditions allow, a spiral CT scan should always be obtained to evaluate concurrent thoraco-abdominal injuries before surgery. Surgical approaches have long been discussed in the literature.[2, 10, 27] One recent systematic review that included 2,023 patients showed that, for acute TDH, an abdominal approach is more frequent than a thoracic one, whereas in the chronic phase, a thoracic approach is more frequent; the requirement to open the second cavity is similar in both approaches.[28] However, this is inconsistent with the results of our study. The choice of approach differs greatly in the literature and depends largely on the first department visited and the preferences of the surgeon. Some authors have noted that a thoracotomy facilitates remission of the intra-thoracic adhesion.[29] In our practice, however, thoracotomy is not used in conjunction with laparotomy, although an extra laparotomy is sometimes needed because necrotic intestines or the ruptured and herniated spleen need to be excised. We recommend a laparotomy for chronic TDH patients with a high possibility of incarceration of herniated viscera because herniated viscera often need to be managed intra-abdominally.[29] Acute TDH patients often have thoraco-abdominal comorbidities of the lung, liver, or spleen, so combined thoracotomy and laparotomy is often necessary for urgent surgical intervention. Some authors have also achieved remarkable success with endoscopic or even robotic repair of the diaphragm, but we do not yet have any experience in this field.[30–33] Meshes are used in the case of hypertonia. In general, re-ruptures are rare, and the best choice of surgical modality depends on concurrent injuries and the preferences of the operating surgeon.

A limitation of this study is its small sample size and retrospective study design, which made it difficult to control the homogeneity between different groups. The small number of penetrating cases may have led to type II error. Patients who died before being admitted to the hospital were not included in this study, which may have introduced selection bias. Meanwhile, given the long time interval between the initial injury and the manifestation of TDH, some first-visit clinical data were not acquired.

## Conclusion

Milder trauma is more likely to result in chronic TDH, a finding that contradicts the published literature. Thoraco-abdominal CT scan should not be excluded for patients with periphrenic injuries and low ISS. Awareness and understanding of diaphragmatic injuries should be improved in trauma centers to avoid delayed diagnosis.

## Supporting information

S1 Data Relevant data underlying the findings described in manuscript are provided. (SAV)

## Abbreviations

TDH: Traumatic diaphragmatic hernia
LOS: Length of the hospital stay
CT: Computed tomography
AIS: Abbreviated Injury Scale
ISS: Injury Severity Score
CR: Chest radiograph
